# Toxicity assessment of doxycycline-aided artificial intelligence-assisted drug design targeting candidate 16S rRNA methyltransferase gene

**DOI:** 10.1186/s40360-025-00875-6

**Published:** 2025-11-21

**Authors:** Hira Mubeen, Nagina Rafiq, Madiha Khan, Saima Jabeen, Muhammad Waseem Shoaib

**Affiliations:** 1 Department of Biotechnology, University of Central Punjab, Lahore, Pakistan; 2 Department of Microbiology, University of Central Punjab, Lahore, Pakistan; 3 Department of Internal Medicine, District Head Quarter, Hospital, Faisalabad, Pakistan; 4 Here General Hospital, Makkah, Saudi Arabia

**Keywords:** 16S rRNA, Methyltransferase, UTI, GI, ADMET, AI

## Abstract

**Background:**

The misfunction of the protein 16SrRNA methyltransferase can result in Urinary tract infections (UTI), Gastrointestinal (GI) infections, sepsis, pneumonia, and wound infections; various tactics are used to lessen the fatal consequences. It confers resistance to aminoglycoside medications, which complicates the treatment of infections caused by these bacteria. Innovative methods are desperately needed to stop these diseases from spreading because there are no reliable medical therapies available.

**Objectives:**

Herein, we aim to evaluate Doxycycline’s Role in AI-Driven Drug Design and identification of effective inhibitors targeting the 16S rRNA methyltransferase gene. Additionally, to investigate the toxicological profiles of designed drug through AI approach for advancement in medical sciences.

**Methodology:**

Methodology involves, selection of three effective de novo medicinal compounds that target the 16SrRNA methyltransferase protein for designing an AI driven drug. Multiple in silico tools were used for designing AI based drug includes: Expasy for protein annotation, ProtParam to calculate physiochemical parameters, SWISS-MODEL to estimate the 3D structure, and UniProt to generate the 16SrRNA methyltransferase protein sequence. An adequate foundation for the development and validation of AI-designed phytochemical medicines for infections is provided by quality assessment, binding site prediction, drug design with WADDAICA, toxicity screening, ADMET evaluation, and docking analysis with CB-dock.

**Results:**

Comprehensive pharmacokinetic and toxicology analyses confirm that the AI-designed doxycycline exhibits a non-toxic character, with particularly high absorption through the blood-brain barrier. Furthermore, the AI-designed doxycycline docked complex demonstrates a strong docking affinity with the 16S rRNA methyltransferase protein, showing a binding energy of approximately − 7.6 kcal/mol, suggesting significant therapeutic potential.

**Conclusion:**

Even though the in silico studies show efficacy and safety, still there is need of in vivo trials to investigate the hidden medical aspects. By addressing existing constraints, presenting a non-invasive approach to infections, and providing viable substitutes for traditional surgical procedures, this work considerably expands the knowledge about newer methods and also helps to understand deep insights of dug design mechanism for treatment.

## Introduction

As an antibiotic, doxycycline belongs to the class of broad spectrum drugs called tetracycline. Through their binding to 16S ribosomal RNA (rRNA) and their inhibition of aminoacyl-tRNA’s ability to attach to the mRNA-ribosome complex, tetracycline is known to decrease bacterial protein production. The precise mechanism of action of these substances is yet unknown, though, because of their activity against other microorganisms like viruses, protozoa, and helminths that lack the 16S rRNA [1]. Moreover, tetracycline is selective enough to largely spare the host organism’s machinery for protein synthesis, even in the face of bacterial and host cell conservation of ribosome structure and function. It is still unclear how the tetracycline specifically suppresses microbial protein production and have such a broad spectrum of effects, despite their lengthy history of use as therapeutic drugs [2].

An in vitro study to correlate ribosomal subunit activity with drug binding revealed that inhibition of tRNA binding to the A-site is solely due to tetracycline cross-linked to the strong binding site on the 30S ribosomal subunit, despite the fact that binding interactions with both the 16S and 23S rRNA had previously been indicated for the tetracycline. Because of this, later research on the tetracycline’s mode of action and how it interacts with ribosomal RNA focused on the 30S ribosomal subunit’s 16S rRNA. However, a recent study found that the tetracycline, minocycline and doxycycline, bind to different synthetic double-stranded RNAs with random base sequences and prevent RNase III from cleaving them in vitro [3]. This suggests that specific base pairs may not be as important for tetracycline binding to RNA as double-stranded secondary structures that are commonly found in cellular RNAs. It also suggests that the tetracycline’ mechanism of action may be related to how the drugs affect the processing of these cellular RNAs [4].

In vivo correlations of this kind may provide valuable information on the mechanisms behind the tetracycline’ broad spectrum of pathogen-opposing activity, as well as their numerous non-infectious therapeutic uses [5].

Of the total cellular RNA in E. coli, ribosomal RNAs make up approximately 95%. Along with serving as enzymes during translation, they construct the ribosomes’ active sites, which are responsible for deciphering the message contained in the mRNA [6]. The increase in bacteria that are resistant to antimicrobial agents poses a serious threat to the control of infectious diseases. This is because illnesses brought on by resistant microbes frequently do not improve with traditional therapy, increasing the chance of death and prolonging the condition. The ability of a bacterium to withstand exposure to an antibiotic is known as antibiotic resistance [7]. Mutations in the bacterial DNA are the main source of antibiotic resistance. Antimicrobial resistance bacteria can grow, disseminate, and endure in environments where antimicrobial medications are misused and used irrationally. Regardless of the degree of the antibiotic’s necessity, resistance to the drug is more likely to develop the longer it is exposed to [8].

The increasing prevalence of antibiotic resistance raises the need for alternative treatments. The number of newly approved medications has been steadily declining, despite a push for innovative antibiotic treatments. Consequently, antibiotic resistance is a serious issue [9]. One of the most often given antibiotics worldwide at the moment is doxycycline, a long-acting, second-generation tetracycline antibiotic that is used to treat a wide range of infectious organisms, including susceptible intracellular and zoonotic infections [10]. It is unknown what the ideal dosage schedule for doxycycline is because it was introduced before pharmacodynamics ideas were understood. With the help of pharmacodynamics data, the best doxycycline dosage schedule was established through this in vitro investigation [11–13]. Phenotypic pathogens such as E. coli and P. multocida, as well as specific Gram-positive pathogens like Streptococcus pneumonia and Staphylococcus aureus, were tested against doxycycline. Two, four, eight, and sixteen times the minimum inhibitory concentration (MIC) of each test organism were represented by different serum concentrations used in time-kill investigations involving each of these organisms [14, 15].

Determine if doxycycline kills susceptible organisms by concentration or time-dependent kinetics was done by measuring the growth of the organisms using colony counts at different intervals throughout a 24-hour period. To find doxycycline’s PAE, research was also conducted [16]. The microorganism utilized was Escherichia coli (A0A8J0KCK0) [17]. The original definition of an antibiotic was a chemical that a single bacteria created that prevented the growth of other microbes. Since the development of synthetic techniques, the definition of an antibiotic has changed, and it is now used to describe a chemical that is produced by a bacterium or a substance that functions similarly and, at low quantities, prevents the growth of other germs. Of all the antimicrobials, antibiotics are one class that poses little threat to the host. Molecular weight less than 2000 characterizes these tiny compounds [18].

Numerous thousands of deaths occur annually due to antibiotic-resistant microorganisms that are widely distributed. The continual rise in microorganism’s resistant to standard antibiotics, especially last-resort medications like vancomycin, is the biggest issue. Resistance genes have the ability to spread quickly over the world, which is concerning since it indicates that this issue is becoming more widespread and calls for international cooperation in order to protect public health. In response to the notable global growth in the population of multidrug resistant strains, the World Health Organization (WHO) identified this issue as a serious threat to global health in 2014 [19]. There has been a widespread implementation of legislative actions aimed at restricting or doing away with the use of antibiotics, both as antibacterial agents in therapy and metaphylaxis (as addressed by EU Regulation 2019/6) and as feed additives for animals. In order to find new antimicrobial agents, identify markers linked to increased innate resistance to pathogens, and ascertain the role of bacteria in the transmission of antibiotic resistance to human and animal microbial flora, measures to reduce drug resistance among microorganisms must also increase research potential in areas like genetic improvement of animals [20].

Strategies for combating antibiotic resistance that are now in use depend on developing next-generation vaccinations and using bacteriophages or their enzymes in an alternate manner. Introducing prebiotics, probiotics, bacterial byproducts, and phytobiotics into animal feeding regimens is another crucial step. Proteases and peptides produced by plants, animals, invertebrates, mammals, and bacteria that have bactericidal properties are also of great interest [21]. Utilizing antimicrobial peptides generated by bacteria that are widely accepted as safe (GRAS), such as Lactobacillus species, Streptomyces, Micrococcus, or yeasts like Saccharomyces and Candidia, is the foundation of this solution [22]. The goal of the conventional fight against antibiotic resistance has been to limit the spread of resistant bacteria and avoid their selection during antibiotic treatment. More recently, the fight against antibiotic resistance has extended to agricultural settings as well. The environment plays a significant role as a source and a conduit in the spread of drug resistance in bacteria, and this understanding has grown in recent years [23].

In order to improve and expedite the drug discovery and development process, machine learning techniques are employed in the quickly developing field of artificial intelligence (AI)-based drug design. By speeding up the identification of possible drug candidates, cutting expenses, and improving the success rate of drug development, it has the potential to completely transform the biotechnology and pharmaceutical sectors. Analyzing biological data, including proteomics, metabolomics, and genomes, to comprehend the disease’s mechanism can assist AI in finding new therapeutic targets. This instance demonstrates the promise and effectiveness of AI-based medication design. Standard drug design processes are more complicated and time-consuming than in silico drug design, validation, and toxicology studies. Using doxycycline as a starting point, this research attempts to create a novel inhibitory medication that inhibits the 16S rRNA methyltransferase protein and deactivates its Hh signaling pathway. The AI-based medication design process made use of the WADDAICA web server. Lipinski’s rule 5 was used to conduct tests in order to assess the drug-like qualities that would make it a viable candidate for pharmaceutical drugs [24]. The Protox II web tool was used to conduct various toxicity studies of tiny compounds.

An effective treatment for the *E. coli* gene is suggested by this study, which may lead to a decreased disease rate. Studies conducted In-silico approaches using computational tools will also provide a useful perspective for adding novel ideas to the literature. The proposed article aims to evaluate the toxicity of doxycycline-assisted artificial intelligence-based drug creation that targets the 16S rRNA methyltransferase gene candidate. This approach may be applied to the development of in vivo medications in the future.

## Materials and methods

### Prediction of primary and tertiary structures of the protein

UniProt (https://www.uniprot.org/) provided the sequence of the targeted 16s rRNA methyltransferase protein [25]. With the use of the SWISS-MODEL the homology modeling and three-dimensional structure of the 16s rRNA methyltransferase protein is predicted. Scientists working in the biological sciences can create protein tertiary structure models with this server’s automated and intuitive method [26].

### Physiochemical properties

Expasy ProtParam, available at https://web.expasy.org/protparam/, was utilized to compute the physiochemical characteristics of the 16s rRNA methyltransferase protein. A tool called ProtParam allows one to compute the input protein’s numerous chemical and physical properties. Included in these determined attributes are the molecular weight, predicted isoelectric point, atomic composition, extinction coefficient, expected half-life, aliphatic index, instability index, and overall average of hydrophilicity (GRAVY) [27].

### Quality prediction of the protein

Combining ProQ with ProSA-web (ProSA-web), the protein quality prediction was carried out. A confidence score derived from statistical and machine learning techniques is provided by the ProQ server, which may be accessed at https://proq.bioinfo.se/cgi-bin/ProQ/ProQ.cgi. It uses a variety of metrics to evaluate protein models [28]. The understanding of protein structure and function is aided by its widespread use in model comparison and validation. The ProSA can be found at https://prosa.services.came.sbg.ac.at/prosa.phpproteins’ structures and functions are predicted and analyzed using a web server. Many structural metrics, including energy profiles, solvation potentials, and Z-scores, are computed in order to evaluate the quality of protein structures [29].

### Validation of the protein structure

PROCHECK, a program on the SAVESv6.0 - Structure Validation Server (ucla.edu) (https://saves.mbi.ucla.edu/), was used to validate the 16s rRNA methyltransferase protein. When examining the protein’s quality and the conformation of amino acid residues inside the protein structure, PROCHECK produces an ERRAT and a RAMACHANDRAN plot that can be used to detect energetically favorable or unfavorable locations. The most advantageous areas are emphasized on this map, along with the extra permitted, liberally permitted, and residue-containing areas that are prohibited [30]. This study offers important new information about the accuracy and consistency of the improved 16S rRNA methyltransferase protein structure.

### Prediction of the binding sites

To view, examine, and work with the protein structure, the Proteins Plus server (https://proteins.plus) was used. In addition to offering resources for molecular modeling, protein engineering, and drug development, Proteins Plus Server is helpful in forecasting and optimizing protein structures. DoGSiteScorer, a program that facilitates the discovery of binding sites on a particular target of interest, is part of the Proteins Plus service. This instrument can detect active areas within a protein structure and analyze protein–ligand interactions [31].

### Drug design by AI

In the context of drug design, researchers can forecast and generate potential drug candidates with the aid of a wide range of computational tools, software, and web servers. These tools often employ molecular modeling, molecular dynamics simulations, virtual screening, and other techniques to examine molecular interactions and identify drugs with possible medicinal efficacy. The goal of the WADDAICA web server is to develop more potent medication candidates by combining deep learning technology with traditional approaches [32]. Deep learning algorithms are used in the WADDAICA initial module (https://heisenberg.ucam.edu:5000/ accessed on June 27, 2024) to scaffold hop molecules in order to modify or create novel, ground-breaking drugs [33].

### Drug likeliness

Pre-clinical drug testing is carried out with the aid of SwissADME (http://www.swissadme.ch/, accessed on June 27, 2024), which helps anticipate the likelihood that target compounds will be developed into pharmaceutical treatments. It forecasted a prospective drug’s critical characteristics, including toxicity, absorption, distribution, metabolism, and excretion [34].

### Toxicity screening

The protox II tool (http://tox.charite.de/protox_II, evaluated on June 27, 2024) was used to anticipate the results of several toxicity analyses [35]. Designed to predict the hazardous effects of tiny chemical compounds, the Pro-tox II tool is a digital laboratory. Predicting the toxicity of these compounds is crucial in the realm of medicine development.

### ADMET and toxicity

This valuable resource, the toxic web server, allows researchers to swiftly and precisely evaluate tiny chemical toxicity profiles. It is based on advanced graph-based signatures and supervised learning, which are available at https://biosig.lab.uq.edu.au/toxcsm/. It outperforms previous methods by producing remarkably accurate prediction models. The target drug candidate produced benefited from the 36 toxicity prediction models provided by this study, which helped create safer medications, insecticides, and herbicides. Toxcsm was employed to evaluate the toxicity of phytochemical substances and produced molecules, and a comparative analysis was conducted [36].

### Docking analysis and validation

One notable molecular docking program is Autodock Vina in PyRx, which may be accessed at https://sourceforge.net/projects/pyrx/. This approach is used to identify the best possible compounds by evaluating how small molecules behave at protein binding sites and forecasting the atomic interactions between the molecules and proteins. The CB-Dock2 version is an improved version of the CB-Dock server that is intended for protein and ligand blind docking. It includes functions that can be accessed at https://cadd.labshare.cn/cb-dock2/php/index.php, including cavity detection, docking, and homologous template fitting. This approach facilitates computer-aided drug discovery by predicting binding sites and affinity between a ligand and protein based on their three-dimensional (3D) structures [37, 38].

### MD simulations

Using the IMods server (http://imods.chaconlab.org/,m, evaluated on September 12, 2024 [39], the docked complex was examined and displayed. Calculating different properties of the docked complex, including the energy required for the complex to degrade, was done through analysis. For the purpose of comprehending drug efficacy, the MD simulation may yield values for the stiffness, Eigen value, and deformability parameters.

## Results

### Prediction of the primary structure of protein

The primary sequence (A0A8J0PCK0_ECOLX) of the uncharacterized 16S rRNA methyltransferase protein (217aa) was obtained from UniProt and belongs to Escherichia coli. The first amino acid was valine and the protein chain finishes at methionine.

### Physiochemical properties of protein

Using Expasy prototyping, the physiochemical characteristics of the 16S rRNA methyltransferase protein have been predicted. The total number of positively charged residues (Arg + Lys) were 31, and its total number of negatively charged residues (Asp + Glu) were 29 with molecular weight 23824.35. Additionally, it is anticipated that the Grand Average of Hydropathicity (GRAVY) will be -0.187 with the Instability Index 37.56. Positive Gravity indicates that this protein is recognized for its hydrophilic nature. The protein’s stability was established by these characteristics.

### Prediction of the tertiary structure of the protein

**Fig. 1:**
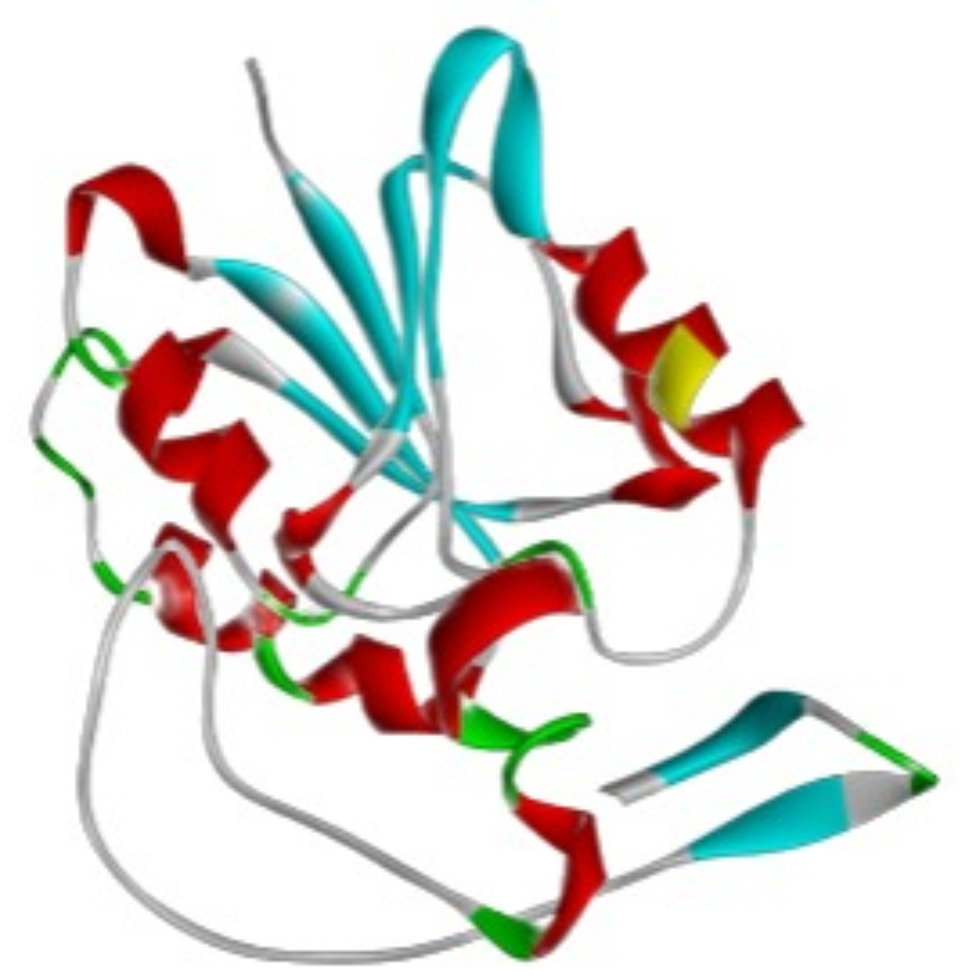
The 3D structure of the 16S rRNA methyltransferase protein predicted using the SWISS-MODEL

Understanding protein function and active sites is made easier by protein 3D structure, which also helps with drug design. As shown in Fig. 1, the Swiss-MODEL has been used to predict the protein’s tertiary structure. Sequence identity and coverage with the template 7ehf.1.A are 100% displayed. The model has a QMEAND and is Co-Global 0.88 ± 0.06 with a Global Model Quality Estimate of around 0.92.

### Quality prediction of the protein

**Fig. 2:**
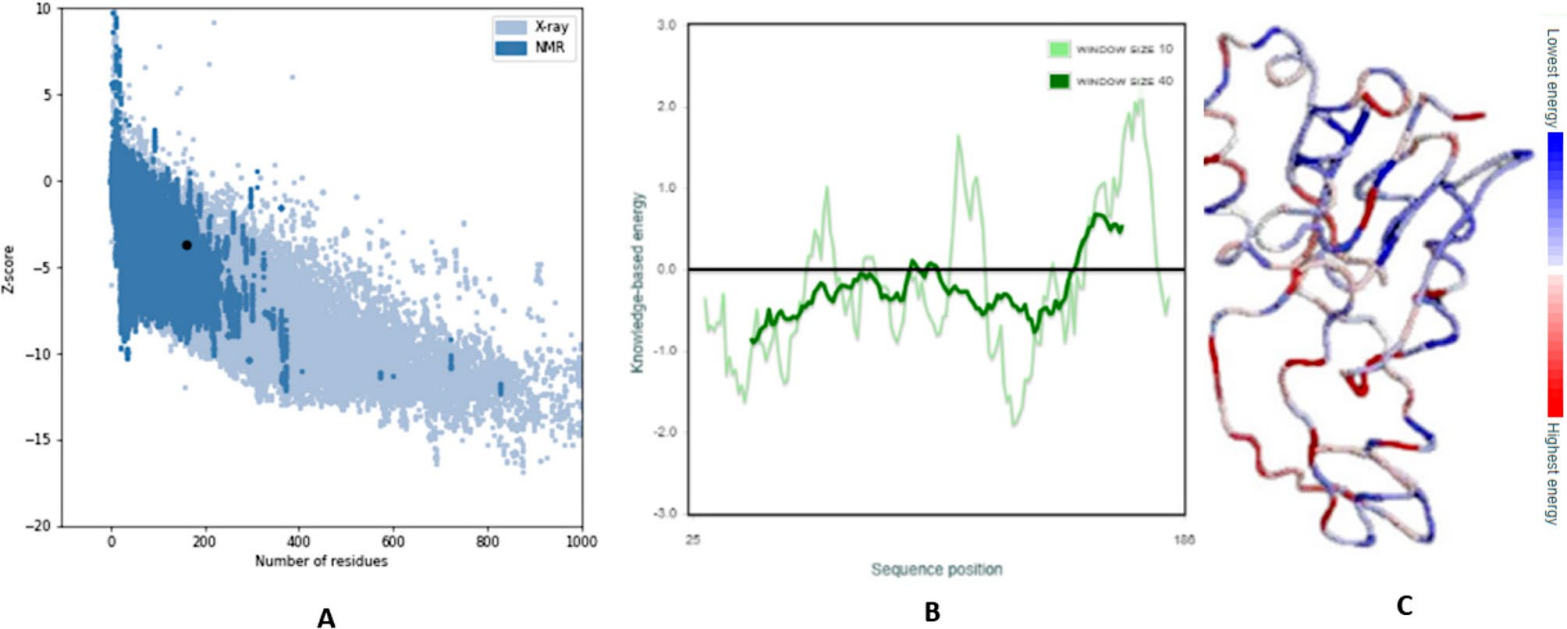
(**a**) Overall quality of the 16S rRNA methyltransferase protein model and (**b**) knowledge-based energy of the local protein model. (**C**) Jmol Cα trace of 1JSQ-A. Residues are colored from blue to red in the order of increasing residue energy

Based on the Z score, the PROSA server provided information about the protein’s quality. The protein is more stable the lower the Z score. The expected Z score for 16S rRNA methyltransferase is − 3.67. Figure 2 also displays the overall quality of the protein tertiary structure, highlighting amino acid residues with larger negative energy levels.

### Validation of the protein

**Fig. 3:**
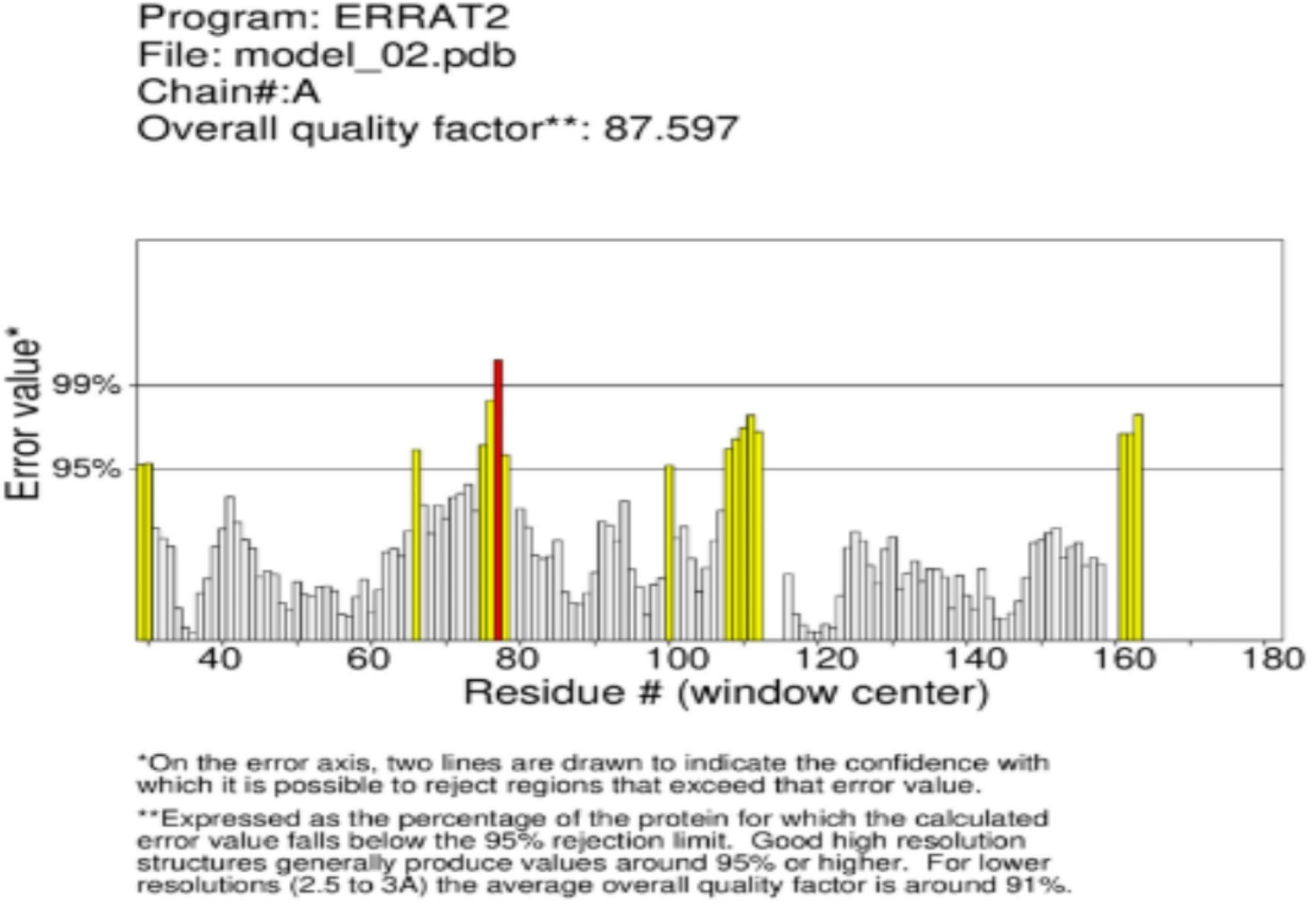
The quality of the protein, predicted by the ERRAT value

**Fig. 4:**
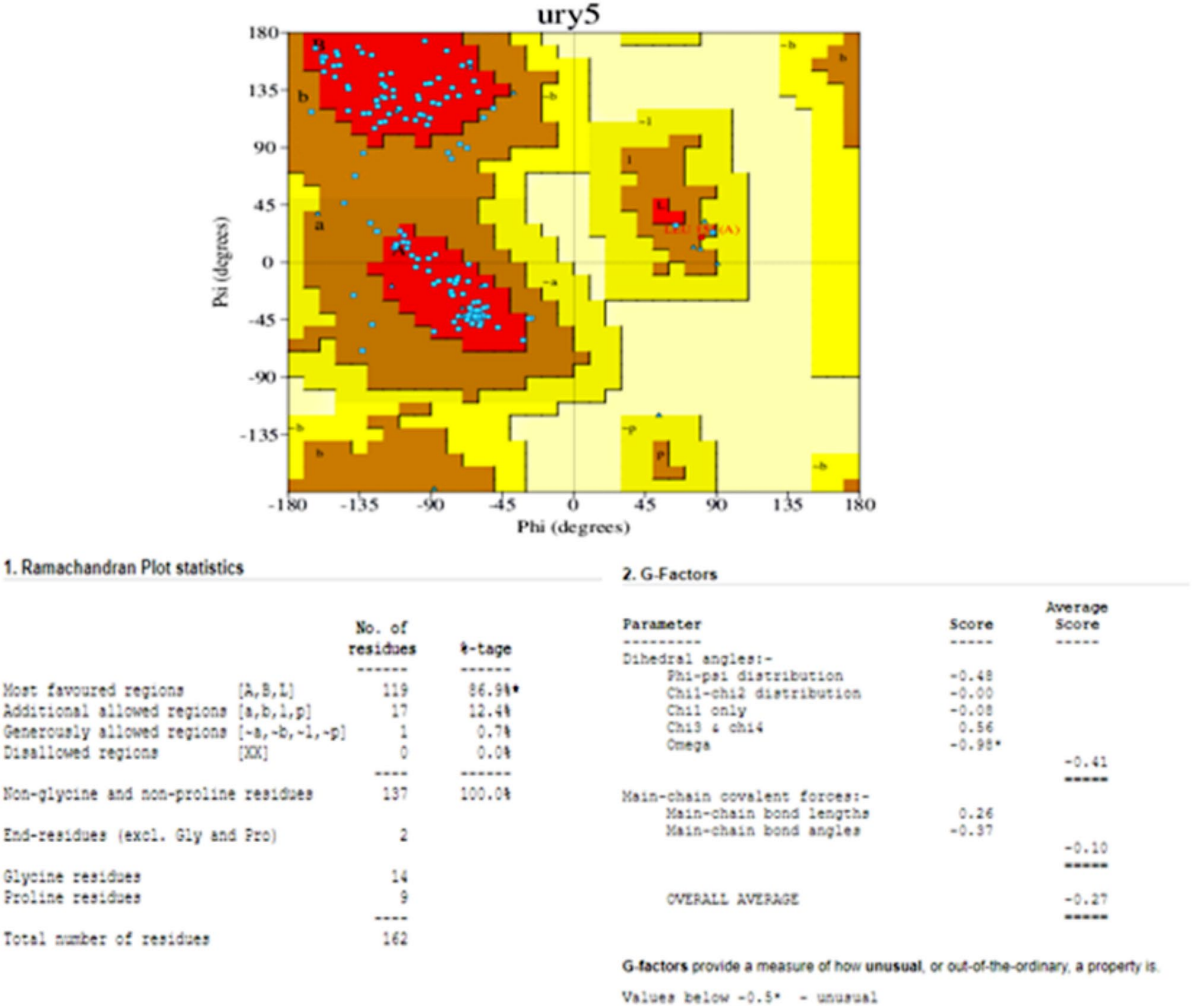
PDBsum analyses for PDB entry ury5. Ramachandran plot from PROCHECK showing the distribution of the protein’s main chain φ-ψ torsion angles (blue squares) relative to the “core” (red) and “allowed” (brown) regions, with residues falling in the “generously allowed” (dark yellow) and “disallowed” (pale yellow) regions plotted as red squares and labeled

PROCHEKC was utilized to predict the validity of the protein. The outstanding structural integrity of the 16S rRNA methyltransferase protein was confirmed by the remarkably high ERRAT value of 87.597, which is a measure of the quality of protein structures. 86.94% of the residues were found in the most preferred locations according to the RAMACHANDRAN plot produced by PROCHECK, whereas 12.4, 0.7, and 0.0% were found in the additional allowed region, generously allowed region, and forbidden region, respectively (Figs. 3 and 4).

### Prediction of binding sites

**Fig. 5:**
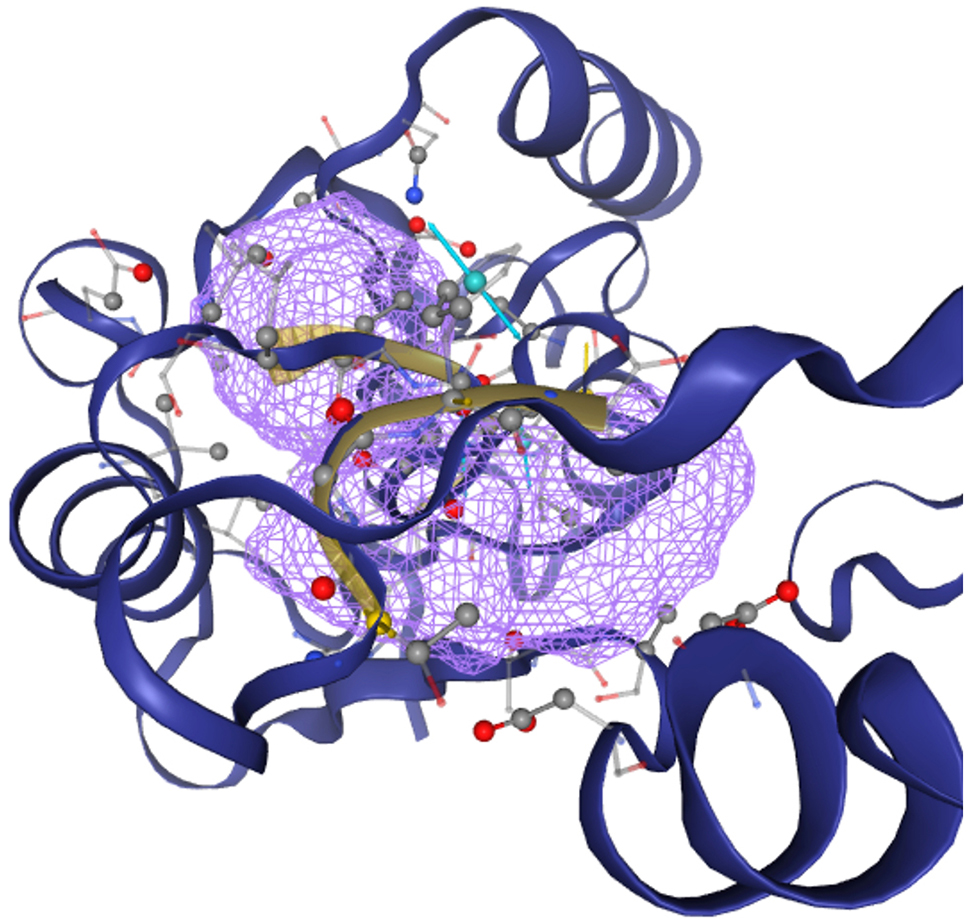
The prediction binding pockets of the 16S rRNA methyltransferase protein. The two active sites are shown in the ball and stick form, and purple color show the grid of the active sites

Figure 5 illustrates the two binding pockets of the 16S rRNA methyltransferase protein that the PROTEIN PLUS predicted. The pocket with the largest volume and surface area, 445.72 Å² and 166.91 Å³, respectively, served as the basis for choosing the active site.

### Drug design by AI

**Fig. 6:**
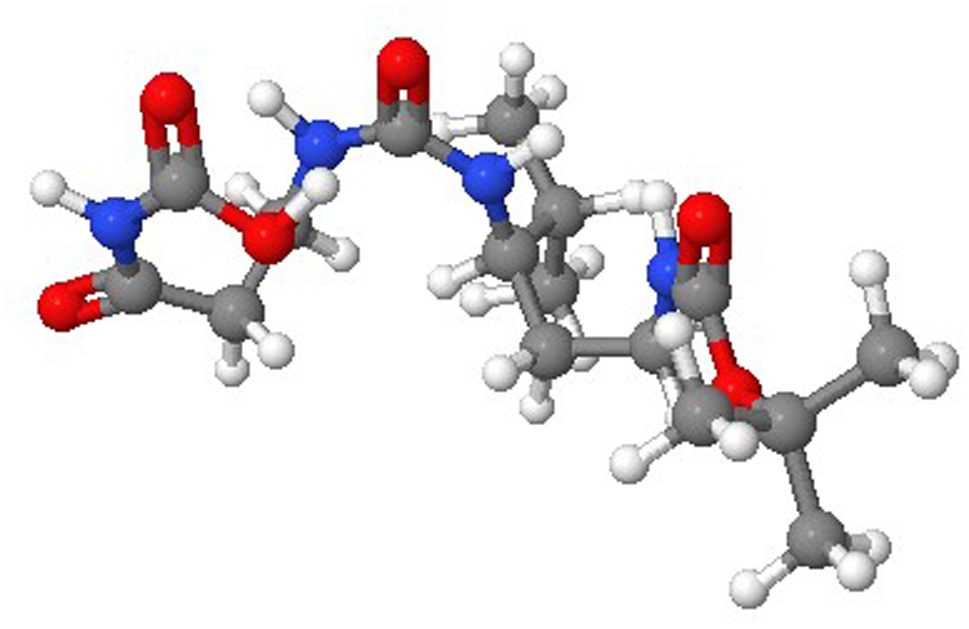
3D structure of molecule 1 of the Doxycycline AI drug designed from WADDAICA

The AI technology utilized doxycycline as a test subject for the WADDAICA platform’s drug design (Fig. 6). Further, it applied the revolutionary deep learning model to transform the naturally occurring chemical into a possible therapeutic candidate. Figure 6 shows the AI-designed chemical.

### Toxicity analysis

The findings indicate a high degree of predictability against neurotoxicity in the doxycycline toxic characteristics of the AI-based compound. Moreover, it was predicted that the substance would not be cytotoxic, mutagenic, or carcinogenic. This Table 1 presents the doxycycline compound’s toxicity profile.

**Table 1:** Toxicity analysis of doxycycline by protox-II

Classification	Target	Shorthand	Prediction	Probability
Organ toxicity	Hepatotoxicity	Dili	Inactive	0.97
Organ toxicity	Neurotoxicity	Neuro	Active	0.52
Toxicity end points	Carcinogenicity	Carcino	Inactive	0.57
Toxicity end points	Immunotoxicity	Immune	Inactive	0.99
Toxicity end points	Mutagenicity	Mutagen	Inactive	0.85
Toxicity end points	Cytotoxicity	Cyto	Inactive	0.78
Tox21-Nuclear receptor signaling pathways	Aryl hydrocarbon Receptor (AhR)	nr_ahr	Inactive	1
Tox21-Nuclear receptor signaling pathways	Androgen Receptor (AR)	nr_ar	Inactive	1
Tox21-Nuclear receptor signaling pathways	Androgen Receptor Ligand Binding Domain (AR-LBD)	nr_ar_lbd	Inactive	0.99
Tox21-Nuclear receptor signaling pathways	Aromatase	nr_aromatase	Inactive	0.82
Tox21-Stress response pathways	Nuclear factor (erythroid-derived 2)-like 2/antioxidant responsive element (nrf2/ARE)	sr_are	Inactive	0.99
Tox21-Stress response pathways	Heat shock factor response element (HSE)	sr_hse	Inactive	0.99
Tox21-Stress response pathways	Mitochondrial Membrane Potential (MMP)	sr_mmp	Inactive	0.83
Tox21-Stress response pathways	Phosphoprotein (Tumor Suppressor) p53	sr_p53	Inactive	0.99
Metabolism	Cytochrome CYP1A2	CYP1A2	Inactive	0.98

### Drug likeliness

**Fig. 7:**
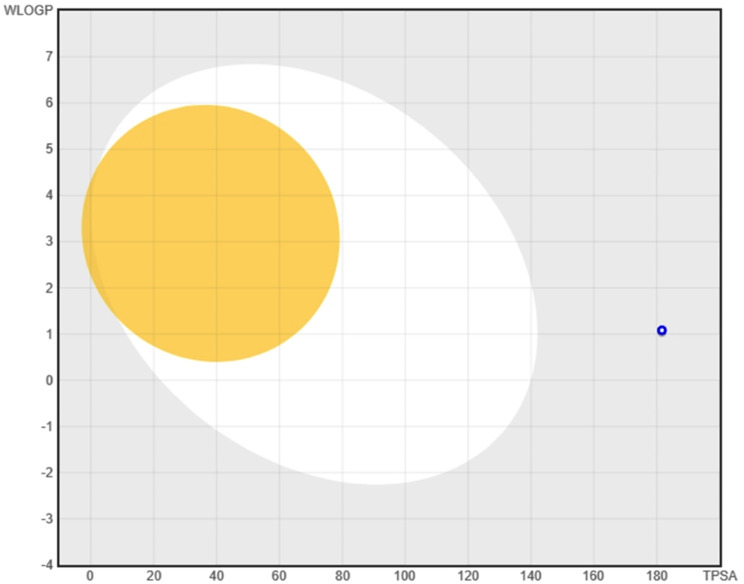
Boiled egg image of doxycycline-aided drug model 1

The results for AI doxycycline for drug likeliness and Physiochemical properties are represented in Table 2 which shows the drug likeliness rules of the AI Fisetin-aided molecule, which were found by utilizing SWISSADME. The SwissADME tool predicted the ADMET characteristics, including log P, mass, donors and acceptors of hydrogen bonds, and molar refractivity. Figure 7 shows the Boiled egg image of doxycycline-aided drug.

**Table 2:** Results of AI doxycycline for drug likeliness, physiochemical properties and their medicinal chemistry

**Drug likeliness**	
Lipinski	Yes
Ghose	No; 1 violation: #atoms > 70
Veber	No; 1 violation: TPSA > 140
Egan	No; 1 violation: TPSA > 131.6
Muegge	No
Bioavailability Score	0.11
**Physiochemical properties**	
Formula	C22H30N2O8
Molecular weight	469.63 g/mol
Num. heavy atoms	32
Num. arom. heavy atoms	0
Fraction Csp3	0.68
Num. rotatable bonds	2
Num. H-bond acceptors	9
Num. H-bond donors	6
Molar Refractivity	111.33
**Medicinal chemistry**	
PAINS	0 alert
Brenk	1 alert: michael_acceptor_4
Leadlikeness	No; 1 violation: MW > 350
Synthetic accessibility	6.67

### Docking results

Using PyRx, which forecasted the outcomes based on cavity identification, docking scores was used to ascertain the interactions between the 16S rRNA methyltransferase protein and Doxycycline ligand. Table 3 illustrates how the phenolic compounds were first screened against the 16S rRNA methyltransferase protein. The maximum binding affinity, − 7.5 kcal/mol, is shown by doxycycline.

**Table 3:** Docking score of compounds

Sr. no	Compounds	2D structure of Ligand molecules	Docking score
1	Doxycycline		-7.5
2	Methacycline		-7.2
3	Ademetionine		-6.8
4	Mitoguazone		-5.7
5	Sardomozide		-5.6

**Fig. 8:**
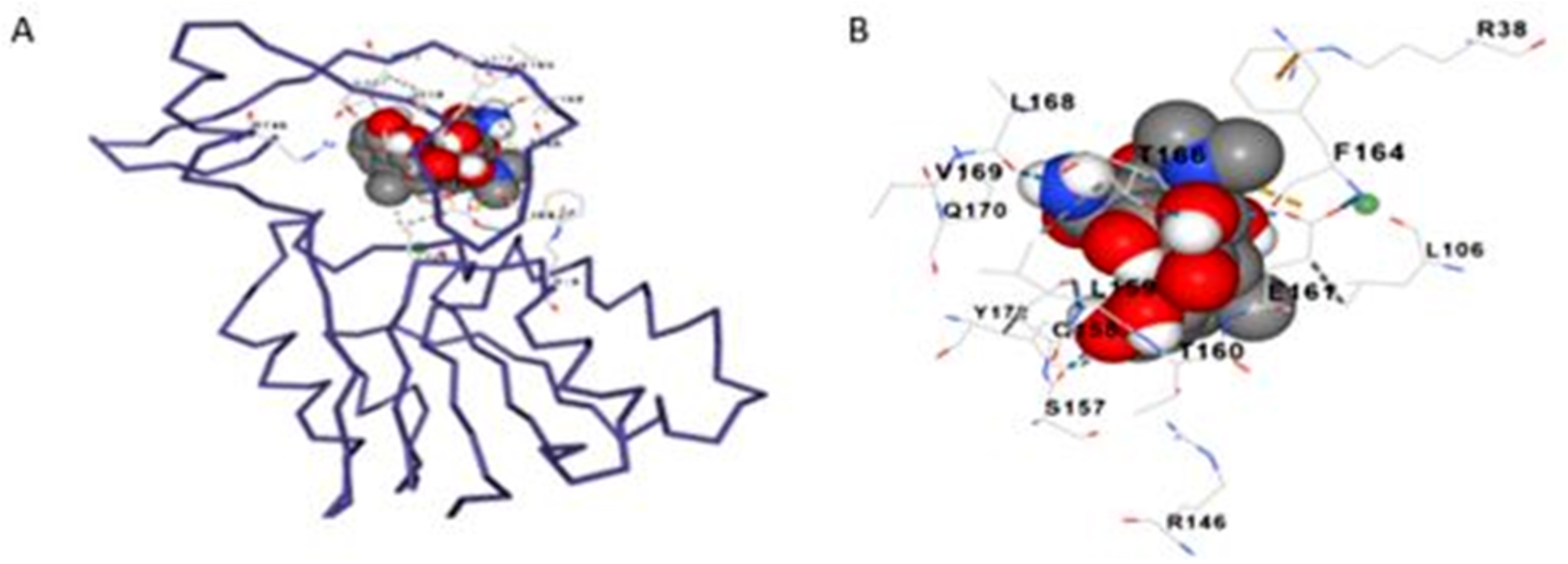
**A**). Dock complex of the 16S rRNA methyltransferase protein and doxycycline designed by CB-Dock server, where gray color structure represents the AI doxycycline ligand. **B**). Protein molecules interacting with the AI drug candidate

Moreover, a CB-dock was used to execute molecular docking between the AI-based Fisetin chemical and the 16S rRNA methyltransferase protein. A dependable binding energy score with a cavity volume of 2558 Å3 is confirmed by the binding affinity of -7.6 kcal/mol observed in the data. Figure 8 (A) displays the protein-ligand dock complex. (ARG38 LEU106 GLY109 SER110 LEU111 ARG113 ALA114 ALA141 ARG146 ASP147 SER157 GLN158 LEU159 THR160 GLU161 GLN162 PHE164 THR166 LEU168 VAL169 GLN170 TYR172 ASN174 ALA186) are the amino acid residues of protein chain A that interact with the doxycycline molecule the most. Figure 8(B) shows how ligands and protein amino acid residues interact.

### Docking results validation

**Fig. 9:**
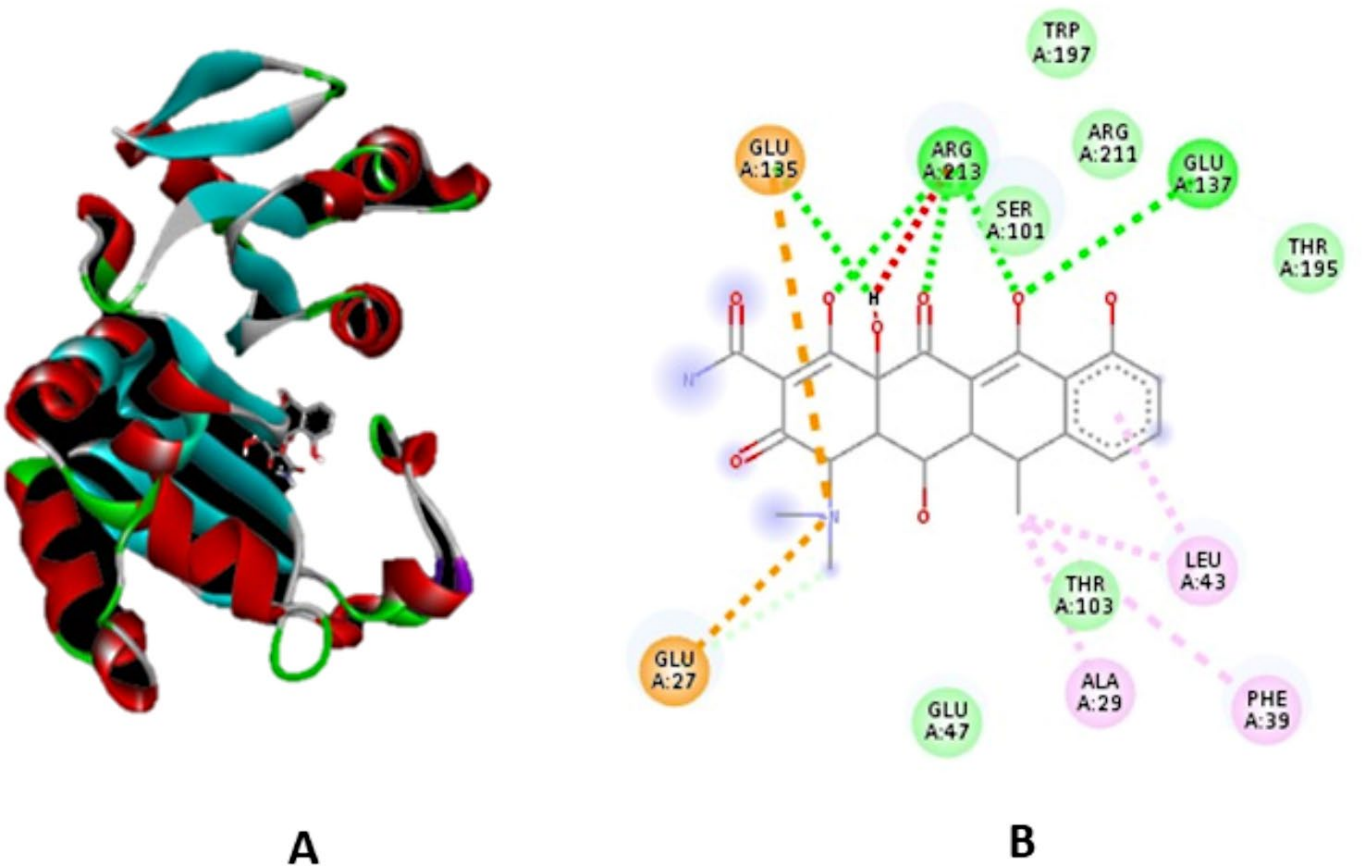
**A**) Dock complex of the 16S rRNA methyltransferase protein and doxycycline designed by Autodock Vina server (**B**) Docking poses of best receptor-ligand complex using Discovery studio

The docking results of the 16S RRNA Methyltransferase protein obtained by the Autodock vina program and the AI drug candidate doxycycline were further confirmed using the shape complementarity docking server Patch dock. Figure 9(A) depicts the complex’s docking, and Fig. 9(B) displays the optimal receptor ligand combination.

### Molecular dynamic simulations

**Fig. 10:**
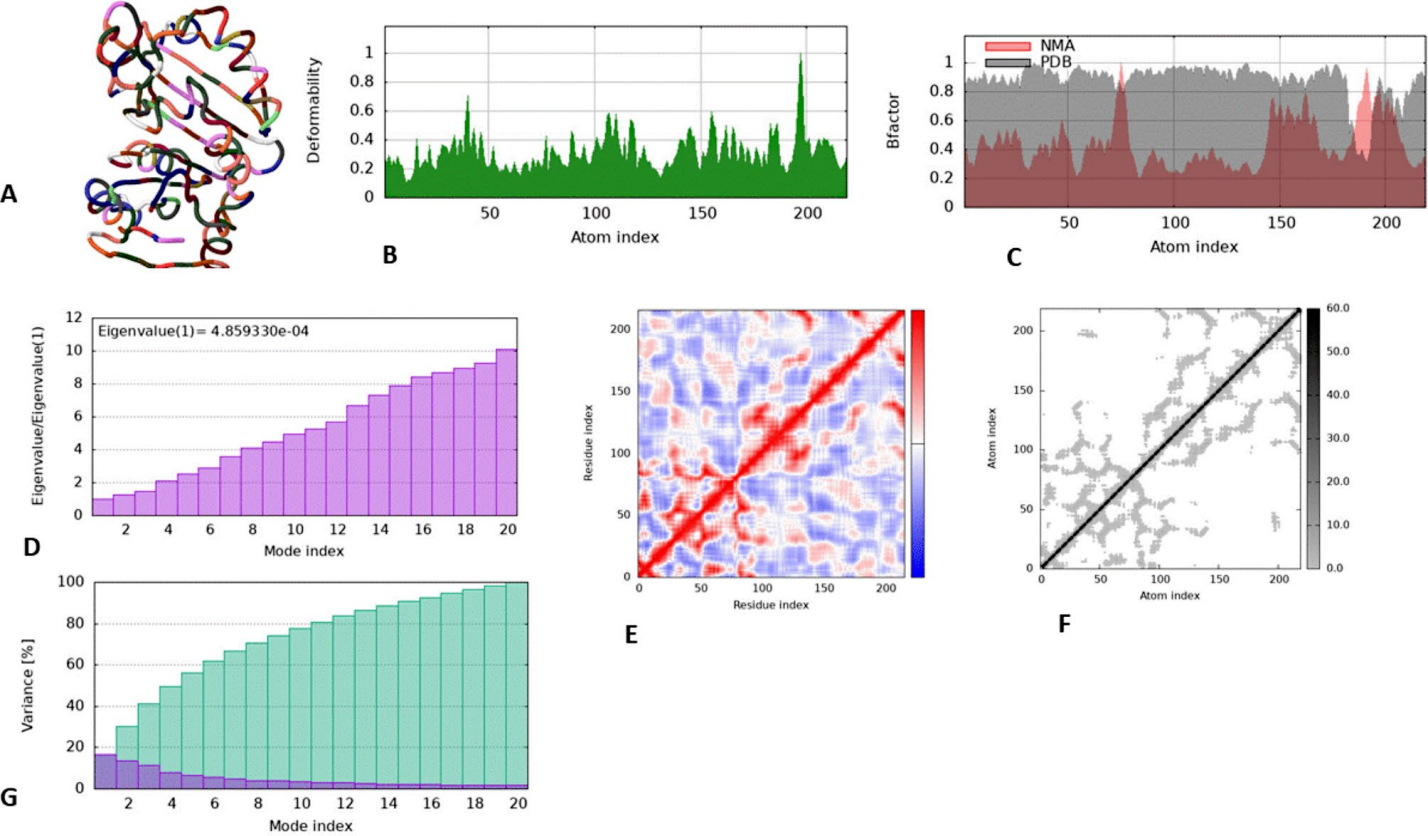
Dock complicated simulations using molecular dynamics. (**a**) Tertiary structure based on MNA mobility. (**b**) The dock complex’s deformability graph. (**c**) The dock complex B-factor graph. (**d**) The dock complex’s Eigenvalues. (**e**) The dock complex’s covariance map, in which the correlated parts are shown in red, the anti-correlated regions in purple, and the uncorrelated regions in white. (**f**) The dock complex’s elastic network, with the stiffest areas displayed in dark gray. (**g**) Mode index of MD simulation

The docked complex of doxycycline and 16S RRNA Methyltransferase was determined and quantified by IMods based on several factors. Here, the results are explained in detail. Different locations were determined to be directly related, as the heat map illustrates. Better interactions between the various residues in the structure are indicated by a low RMSD value. With a lower eigenvalue indicating strong interactions, the docked complex’s estimated eigenvalue was 4.859330e-04. The computed results are shown in Fig. 10.

## Discussion

To release the pre-16S and pre-23 S rRNA for maturation, RNase III cleaves the first lengthy primary ribosomal RNA transcripts/precursors (pre-rRNA) in bacteria. It is not always the nucleotide sequences that determine this cleavage, but rather the double-stranded secondary structures that surround the mature rRNA. There would be an accumulation of pre-rRNA molecules if this cleavage was prevented. RNase III cannot cleave synthetic double-stranded RNAs that doxycycline has been demonstrated to bind to. Doxycycline may consequently prevent normal processing of bacterial rRNA because bacterial rRNA processing mostly depends on RNase III cleavage, which is blocked by the antibiotic [40].

By examining the quantities of distinct rRNA in developing Escherichia coli cells treated with doxycycline, the impact of the drug on bacterial rRNA processing was examined in this work. The findings revealed that mature 16S and 23 S rRNA decreased in a dose-dependent manner when the first rRNA transcripts and lengthy precursors accumulated. At low drug concentrations, treated cells exhibited morphological elongation; at higher drug concentrations, nucleoid degeneration, a sign of cell death, transpired [41].

In the event that RNase III cleavage is not present, 16S rRNA could nevertheless mature, as evidenced by the growth of RNase III-deficient bacteria. It’s thought that this is because other nucleases working without RNase III provide an alternate processing route [42].

Prokaryotes share a highly conserved rRNA processing mechanism via the RNase III cleavage pathway, which accounts for the doxycycline’ broad spectrum of antibacterial action [43]. However, a far more intricate route that is independent of RNase III is involved in the digestion of ribosomal RNA in eukaryotes. Furthermore, ionic conditions—particularly those related to Mg2 + and divalent metal ion concentrations—are unfavorable for doxycycline binding during eukaryotic rRNA processing, which takes place in a protected habitat called ribosomes [44, 45]. Protein synthesis in bacteria may be selectively inhibited due to variations in the processing pathways of prokaryotic and eukaryotic ribosomal RNAs, with eukaryotic protein synthesis being less affected. The bacteriostatic mechanism of action of tetracycline is supported by the drug’s gradual recovery from its inhibitory effects on the maturation of ribosomal RNA [6].

Monotherapy, such as β-lactams or carbapenem, for serious infections has become useless and unsuccessful due to the widespread emergence of pan- and multidrug-resistant bacteria. In the treatment of infectious diseases today, medication combination therapies combining various antibiotics are indispensable. A class of antibiotics known for their broad antimicrobial spectrum that includes the ability to inhibit both Gram-negative and some Gram-positive bacteria, aminoglycosides were among the first to be discovered and utilized in therapeutic settings [46].

Aminoglycoside antibiotics are once again the center of attention in the clinic as one of the best choices for medication combination therapy [47].

However, resistance to aminoglycosides in bacteria is caused by a range of resistance mechanisms. The synthesis of 16S-RMTases is one of the pathways that could be dangerous. The rationale behind this assertion is their ability to induce not only total resistance to a wide variety of aminoglycosides, but also high-level resistance that is unaffected by dosage increases. Additionally, a number of studies have demonstrated that corresponding genotype subtypes frequently result in higher levels of resistance to aminoglycosides; for instance, rmtF2 can mediate resistance to arbekacin and amikacin with MIC > 1024 µg/mL [48].

In order to address the current state of aminoglycoside resistance and find the best screening practices and techniques for quickly identifying strains expressing 16S-RMTase, it will be crucial to lobby clinicians and researchers in the future. Evaluation of the molecular epidemiology of aminoglycoside resistance genes and thorough examination of associated mobile genetic elements are also strongly advised in order to manage the occurrence and worldwide spread of multidrug-resistant bacteria [49].

As of right now, reports indicate that tetracycline binding prevents RNase III from digesting dsRNA [8]. RNase III is primary to the digestion of bacterial rRNA. It is becoming increasingly apparent that the 16S rRNA binding mechanism currently held for the antibacterial action of tetracycline is not only limited but also does not explain their activities against other, nonbacterial, pathogens and under certain pathological conditions. It has therefore become imperative to consider alternative binding sites/modes that may offer insights into the broad spectrum of activity and antimicrobial selectivity of tetracycline. The binding of the tetracycline to cellular dsRNAs and the consequent effects on affected RNA processing and function could be a worthwhile alternative to explore [50].

The lethal effects of the doxycycline medication on the growing cells were described using a robust model. In order to validate the delivery of doxycycline as a highly hazardous treatment using the 16S rRNA methyltransferase protein, this study also included a computer assessment of hepatotoxicity and other toxicities. But to conduct this study, which employed important AI-based deep learning algorithms to create the medication, the WADDAICA web server was assessed. Docking is a crucial screening technique because it makes it possible to virtually predict how small molecules, like medications, will interact with proteins, such receptors or enzymes. This simulation can provide valuable insights into the binding affinity, protein–ligand interaction energetics, and orientation without requiring experimental techniques. A scoring function is chosen based on the specific scenario at hand and the desired trade-off between speed and accuracy. Remembering that various scoring functions could have unique advantages and disadvantages is crucial. Other scoring functions might be more suitable in different situations, even though Dock Thor Vina might work well for a certain use scenario. It is always a good idea to test and evaluate different scoring functions in order to determine which one best suits a certain scenario.

For the AI-based medication design, doxycycline was chosen as the component de novo molecule to be recovered against the 16S rRNA methyltransferase protein. Due to its active prediction of neurotoxicity, doxycycline is extremely dangerous, as evidenced by the comparative study of its toxicity with 16S rRNA methyltransferase.

The findings are further validated by the comparison of the ADMET study. When the AI-designed doxycycline molecule was docked with 16S rRNA methyltransferase, the binding affinity of − 7.6 kcal/mol was obtained. This indicates that the AI-created molecule is more effective because its toxicities analysis is inactive, and we can therefore take it into consideration as a possible drug candidate against rRNA processing. By utilizing Patchdock to verify the form complementarity of the ligand and protein, the docking results were further validated.

## Conclusion

To explore the hidden medical features, in vivo trials are still necessary, even when in silico research demonstrate efficacy and safety. This work contributes significantly to the understanding of deeper insights of dug design mechanism for treatment, while also addressing current constraints, presenting a non-invasive approach to infections, and offering workable alternatives to conventional surgical procedures.

## Data Availability

No datasets were generated or analysed during the current study.
